# Comprehensive and Rapid Real-Time PCR Analysis of 21 Foodborne Outbreaks

**DOI:** 10.1155/2009/917623

**Published:** 2009-06-24

**Authors:** Hiroshi Fukushima, Kazunori Katsube, Yoshie Tsunomori, Ryoko Kishi, Junko Atsuta, Yuko Akiba

**Affiliations:** Shimane Prefectural Institute of Public Health and Environmental Science, 582-1 Nishihamasada, Matsue City, Shimane 690-0122, Japan

## Abstract

A set of four duplex SYBR Green I PCR (SG-PCR) assay combined with DNA extraction using QIAamp DNA Stool Mini kit was evaluated for the detection of foodborne bacteria from 21 foodborne outbreaks. The causative pathogens were detected in almost all cases in 2 hours or less. The first run was for the detection of 8 main foodborne pathogens in 5 stool specimens within 2 hours and the second run was for the detection of other unusual suspect pathogens within a further 45 minutes. After 2 to 4 days, the causative agents were isolated and identified. The results proved that for comprehensive and rapid molecular diagnosis in foodborne outbreaks, Duplex SG-PCR assay is not only very useful, but is also economically viable for one-step differentiation of causative pathogens in fecal specimens obtained from symptomatic patients. This then allows for effective diagnosis and management of foodborne outbreaks.

## 1. Introduction

The introduction of real-time PCR in foodborne outbreak investigations provides an opportunity for rapid detection of pathogens in food and clinical settings [1]. The benefits to public health administration from rapid real-time PCR assays are most notable after comprehensive and rapid detection of bacteria. The results can quickly inform a public health administrator about the causative pathogens of foodborne outbreak, allowing a more accurate, effective, and timely response. Abubakar et al. [2] implied in the Health Technology Assessment program (now part of the National Institute for Health Research, UK) that the feasibility of conversion to rapid methods such as multiplex PCR and DNA microarrays is dependent on localized considerations, including the community prevalence rates for specific pathogens, the skill base, and subsequent training costs for laboratory staff and spare capacity available to ensure adequate laboratory space for new equipment. Although these tests look promising, further studies are necessary to assess their usefulness [2].

Apart from saving time, real-time PCR is sensitive, highly specific and offers the potential for quantification [3]. The risk of cross-contamination is significantly reduced, and high-throughput performance and automation are possible since no post-PCR manipulations are required [4]. In principle, two different chemistries are available for real-time detection of PCR products: fluorescent probes that bind specifically to certain DNA sequences and fluorescent dyes that intercalate into any double-stranded DNA. Fluorescent-probe based real-time PCR (TaqMan PCR) studies to detect causative pathogens from foodborne outbreaks in feces using TaqMan probes have been carried out [3–6]. TaqMan PCR assays require the availability of primers and probes that must be selected according to very rigid criteria. Use of simple, cheaper double-stranded DNA-binding dye SYBR green I for detection of PCR amplicons (SG-PCR) overcomes this limitation. Therefore, real-time PCR could be applied without the need for fluorescent probes [7]. In the absence of probes, the specificityof the reaction is determined on the basis of the melting temperature (*T*_*m*_). The advantages of SG-PCR over TaqMan PCR include the relative simplicity and reduced cost of SYBR Green I compared to TaqMan probes [8]. Recently, the application of SG-PCR for the detection of foodborne bacteria in different samples has been increased [1, 9–12]. Duplex SG-PCR assays have been carried out to detect causative bacteria in feces from foodborne outbreaks [4, 10, 13].

We previously reported a set of four duplex SG-PCR assays for one-step differentiation of 8 genes of foodborne pathogens in DNA extracted from 5 feces using 32 capillary tubes of LightCycler (Roche). The first run was for the detection of 8 main foodborne pathogens and the second run was for the other pathogens. We reported here that improved diagnostic duplex SG-PCR assays were upgraded with new highly sensitive primer pairs for 11 foodborne pathogens. These assays successfully identified the causative pathogens of foodborne outbreaks caused by enteropathogenic *Escherichia coli*, enterohemorrhagic *E. coli*, *astA-*positive *E. coli, Plesiomonas shigelloides, Vibrio parahaemolyticus, Campylobacter jejuni*, *Clostridium perfringens*, *Bacillus cereus*, or *Staphylococcus aureus* in 21 cases from 2002 to 2007. This assay is simple, rapid, inexpensive, reliable as well as suitable for comprehensive, rapid detection of causative pathogens in foodborne outbreaks.

## 2. Material and Methods

### 2.1. Bacterial Strains

 The 27 foodborne bacteria used in this study are *E. coli* (enteroinvasive *E. coli* (EIEC), enteropathogenic *E. coli* (EPEC), enterohemorrhagic *E. coli* (EHEC), enterotoxigenic *E. coli* (ETEC), and enteroaggregative *E. coli* (EAEC)), *Shigella* *sonnei*, *Salmonella* Enteritidis, *Yersinia enterocolitica*, *Yersinia pseudotuberculosis*, *Providencia alcalifaciens*, *Plesiomonas shigelloides*, *Campylobacter jejuni*, *C. coli*, *Vibrio cholerae*, TDH-positive *V. parahaemolyticus*, TRH-positive *V. parahaemolyticus*, *Aeromonas hydrophila*, *Staphylococcus aureus*, emetic *Bacillus cereus,* enterotoxigenic *B. cereus*, and *Clostridium perfringens* (Table 1). Bacterial cultures and viable-cell counting were described in a previous report [10]. For template DNA of each foodborne pathogen as a PCR control, 200 *μ*L of each bacterial culture (10^8^ CFU/mL) was treated with a QIAamp DNA Stool Mini kit (Qiagen) in the same procedure as the following stool treatments.

### 2.2. Primer Design

The 22 primer pairs used in this study for the detection of *E. coli* (EIEC, EPEC, EHEC, ETEC, and EAEC), *Salmonella* *enterica*, *Shigella* spp., *Y. enterocolitica, Y. pseudotuberculosis, P. alcalifaciens, C. jejuni, C. coli, V. cholerae, V. parahaemolyticus, A. hydrophila*, *P. shigelloides, S. aureus, C. perfringens*, and *B. cereus* were described in our previous reports [10, 13] for cases 1 to 19. The newly designed 22 primer pairs listed in Table 2 were used for cases 19 to 21. In this study, 10 primer pairs (marked with * in Table 2) were newly designed or selected from earlier publications (see Table 2 references). The 4 primer pairs (ces, yadA-X, CCceuE, and aggR-Z) were newly designed. The ces primer was constructed from cereulide synthetase gene of emetic *B. cereus* [4], the yadA-X primer from *yadA* gene on the plasmid present in virulent *Yersinia* spp. [24], the CCceuE primer from *ceuE* gene encoding of a lipoprotein component of a binding-protein-dependent transport system for the siderophore enterochelin of *C. coli* [25], and the aggR-Z primer from *aggR* gene encoding of a transcriptional activator for EAEC aggregative adherence fimbria I expression [26]. To determine the specific primers ces, yadA-X, CCceuE, and aggR-Z, the genes of *ces, yadA, ceuE*, and *aggR* that were expected to be unique were selected with the Basic Local Alignment Search Tool (BLAST) program within GenBank and were designed by Biosearch Technologies Inc. (USA). Other primer pairs were those used in earlier publications (see Table 2 references). All oligonucleotide primers were synthesized by Invitrogen (Yokohama, Japan) or Biosearch Technologies Inc. (USA).

### 2.3. Duplex SG-PCR with Feces

Feces (1 g) from 5 patients were weighed aseptically from the mass sample collected for virological inspection, placed into sterile tubes, and homogenized with 9 mL of distilled water. Then, 200 *μ*L of stool suspension was treated with a QIAamp DNA Stool Mini kit. For real-time PCR, we used SYBR *Premix EX Taq* (Takara, Japan), 32 glass capillary tubes, and a LightCycler instrument (Roche Diagnostics, Mannheim, Germany) as described by the manufacturer. Duplex SG-PCR was performed using 32 glass capillary tubes with 4 groups of 2 primer sets on the LC instrument for each run. Analysis of each group of primer pairs was made in 8 glass capillary tubes; each of which included 1 negative DNA control consisting of PCR-grade water, 2 positive controls, and template DNA from 5 feces. The first run of duplex SG-PCR was analyzed using 4 primer sets selected from 11 primer sets described in our previous reports [10, 13]. The newly first run primer set including eae plus FemB, AB plus EAST1, Tdh plus Ces-TM, and Styinva plus GAP (see Table 2) was used for analysis of cases 19 to 21. The second run was analyzed using 4 primer sets selected from the following primer sets: LT plus AHH1, STa plus PSG, aggR-Z plus virA, SG plus PAG and the third run using yadA-X plus CCceuE, and hlyA plus Trh. The *eaeA*-positive samples were analyzed by simple PCR using primers JMS1 and JMS2. Each reaction tube contained 10 *μ*L of SYBR *Premix EX Taq*, 6.8 *μ*L of PCR-grade H_2_O, 0.4 *μ*L of both forward and reverse primers (10 *μ*M) for the target gene of two foodborne pathogens, and 2 *μ*L of template DNA in a 20 *μ*L PCR mixture. The assay cycling profile was 95°C for 10 minutes, followed by 30 cycles of denaturation at 95°C for 5 seconds and then annealing at 60°C for 20 seconds. Fluorescence signals were measured once per cycle at the end of the extension step. After PCR amplification, a melting temperature curve analysis was done. Next, the LightCycler PCR products were cooled to 65°C and then heated to 95°C at a rate of 0.1°C per second. The fluorescence signals obtained were continuously monitored to confirm amplification specificity during 1 hour of analysis. The products' melting temperature peaks were calculated by performing 10 or more assays per sample and were based on the initial fluorescence curve found by plotting the negative derivative of fluorescence over temperature versus temperature. To quantify target bacteria in feces, DNA samples extracted with the QIAamp DNA Stool Mini kit from target bacteria were used to form a standard curve. Two microliters of a serial 10-fold dilution of DNA (Easy Dilution from Takara, Japan) were prepared and analyzed under the conditions specified above.

### 2.4. Duplex SG-PCR Analysis in 21 Foodborne Outbreaks

21 foodborne outbreak cases examined by duplex SG-PCR in Shimane Prefecture, Japan from 2002 to 2007 are shown in Table 3.

## 3. Results and Discussion

### 3.1. Duplex SG-PCR Procedures

**Figure 1:**
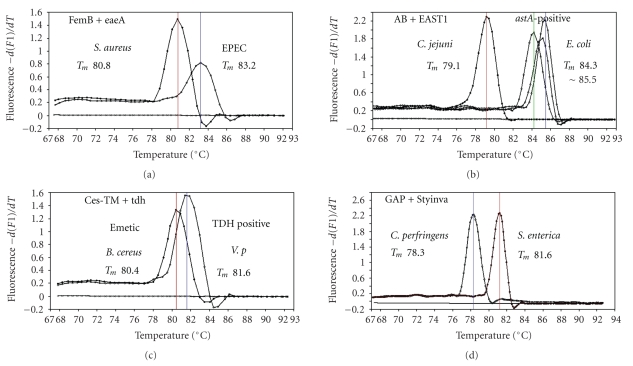
Melting curve analysis of duplex SYBR Green I PCR products in the first run using four primer sets: FemB plus eaeA, AB plus EAST1, ces plus tdh, and GAP plus Styinva.

**Figure 2:**
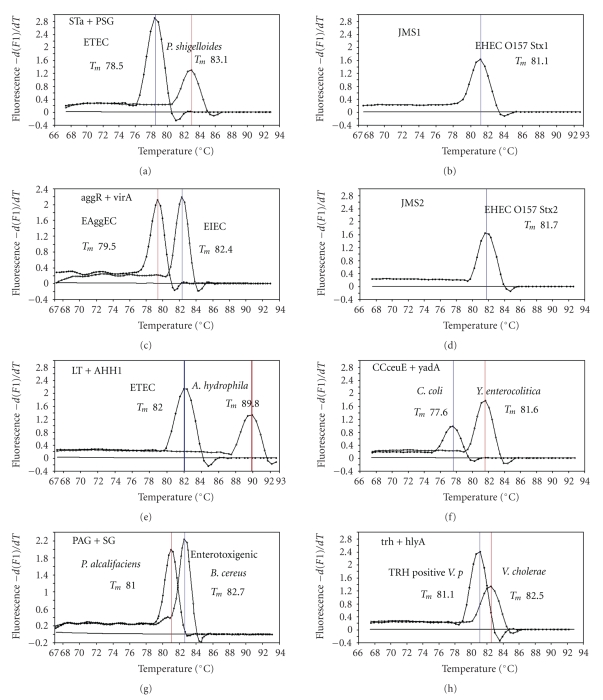
Melting curve analysis of duplex SYBR Green I PCR products in the second run using four primer sets: ST plus PSG, aggR plus virA, LT plus AHH1, and PAG plus SG; the third run using two primer sets: CCcesE plus yadA and trh plus hlyA; simple PCR with primers JMS 1 and JMS2.

We previously reported duplex SG-PCR assays for detection of 19 species of foodborne pathogens using 22 primer pairs [10, 13]. After that, more accurate duplex SG-PCR assays were designed by 10 more sensitive and specific primers including 6 primers (FemB, AB, ces-TM, Styinva, SG, and AHH1) selected from earlier publications (see references in Table 2) and 5 new primers (eae, aggR-Z, yadA-X, and CCceuE) constructed in this study. The new primer set was used for cases 19 to 21. Real-time SG-PCR procedures using 22 primer pairs for the detection of 15 bacterial species, including 5 *E coli* subgroups, were developed for the duplex assay. The primer sequence, target, SG-PCR product size, *T*_*m*_ values (mean plus standard deviation from a range of 10 assays), specificities, and references are summarized and listed in Tables 1 and 2. The primer virA detects *virA* gene of *Shigella* spp. and EIEC; the primer eae detects *eaeA* gene of EPEC and EHEC, and the primer EAST-1 detects *astA* gene of EAEC and ETEC. Primer hlyA detected *hlyA* gene of *V. cholerae* strains O1 and O139 as well as non-O1 strains. The primer SG for the detection of *nheB* (nonhemolytic enterotoxin B) gene of *B. cereus* cross-reacts with enterotoxigenic and emetic strains and the primer ces-TM detects cereulide synthetase gene of emetic strain of *B. cereus.* The *nheB* and *ces* gene positive strains were identified with emetic strains and the *nheB* gene positive and *ces* gene negative strains with enterotoxigenic strains. A new primer yadA-X for *Yersinia* adhesion reacts with virulent *Y. enterocolitica* and *Y. pseudotuberculosis,* but not with nonpathogenic strains of *Yersinia* spp. (data not shown). Other primers, including new primers aggR-Z and CCceuE, specifically detect each gene of EAEC and *C. coli*. Food-borne Outbreak Investigation Report (http://www.mhlw.go.jp/topics/syokuchu/), Ministry of Health, Labor and Welfare, Japan during 2005 to 2007 shows that 97% of foodborne outbreaks were caused by the following 7 species of foodborne pathogens: *S. enterica* (58.3%), *C. jejuni* (15.2%), TDH-producing *V. parahaemolyticus* (8.3%), *S. aureus* (7.2%), *C. perfringens* (3.6%), emetic *B. cereus* (1.6%), EHEC (2.9%), and other virulent *E. coli* (2.1%) which include *astA*-positive *E. coli* which is a strain of *E. coli* that does not possess any diarrheagenic characteristics except the EAEC heat-stable toxin 1 (EAST1) gene and is frequently isolated in diarrhea outbreaks [27]. Using of 4 primer sets of 2 primer pairs, including newly selected or designed 6 primer pairs, for the detection of 7 main foodborne pathogens and *astA-*positive *E. coli* in the first run of duplex SG-PCR brought out the comprehensive, rapid, and sensitive detection of causative pathogens in foodborne pathogens to cases 19 to 21 (Table 2 and Figures 1 and 2). The second run of duplex SG-PCR used 4 primer sets and the final run utilized 2 primer sets selected from the remaining 4 primer pairs. The primers JMS1 and JMS2 were used for the single PCR detection of *stx1* and/or *stx2* genes from the *eaeA* gene-positive samples for the confirmation of EHEC. Figures 1 and 2 show the *T*_*m*_ curves of the duplex SG-PCR products of the template DNA samples in each run. In duplex SG-PCR assay with two primer pairs, each PCR product was generated with a different *T*_*m*_ curve. These could be resolved in a LightCycler by using *T*_*m*_ curve analysis when a target bacterium was present in the reaction tube.

### 3.2. Using Duplex SG-PCR for Identification of the Causative Agent in 21 Foodborne Outbreaks

Table 3 shows epidemiological and clinical investigations in 21 foodborne outbreaks examined by duplex SG-PCR analysis in Shimane Prefecture, Japan from 2002 to 2007. From samples of feces, we used a combination of duplex SG-PCR assay with DNA extraction using a QIAamp DNA Stool Mini kit. The SG-PCR assay is rapid, specific, and sensitive as a detection technique. The DNA extraction of 5 stool specimens with the QIAamp DNA Stool Mini kit was carried out within 1 hour and it effectively removed inhibitors present in feces. The duplex SG-PCR assay was also carried out within 1 hour. The 7 species (listed previously) of foodborne bacteria, which included 3 groups of *E. coli*, were detected from 111 (58.1%) of 191 feces in 21 cases by duplex SG-PCR. Then these causative agents were isolated and identified after 2 to 4 days. With the exception of two cases (cases 10 and 11), the first run of duplex SG-PCR confirmed the presence of a pathogen in 54 (58.1%) of 93 feces in 19 (90.5%) cases within 2 hours. The exceptions were case 10 where a confirmation test was necessary to detect the *eaeA* gene of EHEC O26 and case 11 where *astA*-positive *E. coli* was detected on the third run. In the first run, DNA samples extracted from 5 feces (1, 3, 4, or 7 feces in 6 cases) of symptomatic patients were used and the causative pathogens were detected from 1 to 5 samples: 1 (in 8 cases: 1, 2, 4, 7, 8, 15, 19, and 21), 2 (in 3 cases: 9, 13, and 20), 3 (in 3 cases: 16, 18, and 21), 4 (in 3 cases: 5, 6, and 17), and 5 samples (in 3 cases: 3, 12, and 14). Then the causative pathogens were later isolated in a routine laboratory. In cases 11 and 12, *C. perfringens* or *C. jejuni* was detected by duplex SG-qPCR with more than 10^5^ CFU/g feces from only 1 sample and *C. perfringens* was then also isolated from only 1 of 46 samples and *C. jejuni* from only 1 of 16 samples by culture method. Therefore, the infections with both these pathogens were determined to be sporadic cases and they were immediately eliminated as causative pathogens in cases 11 and 12. It was confirmed that duplex SG-PCR analysis of 5 feces collected from symptomatic patients was ultimately the most effective screening method for foodborne pathogens in foodborne outbreaks [10, 13].

Duplex SG-PCR rapidly and accurately demonstrated that 12 (57.1%) of 21 cases were caused with a single foodborne pathogen such as *C. jejuni* (6 cases), *C. perfringens* (3 cases), *B. cereus* (2 cases), and TDH-producing *V. parahaemolyticus* (one case). There were also 7 (33.3%) cases with plural foodborne bacterial pathogens (such as *astA*-positive *E. coli,* EPEC, *C. jejuni*, *C. perfringens, A. hydrophila*, and *P. shigelloides*) and 2 (9.5%) cases with foodborne bacterial pathogens (*astA*-positive *E. coli* or EHEC O:26) and norovirus. In cases 2 and 10, although detection of norovirus is out of the scope of our work, norovirus and foodborne bacterial pathogens were concomitantly detected by conventional PCR analysis in our virological laboratory. In case 2 in which norovirus was detected in 6 of 7 feces, the *astA* gene of EAEC was detected from 7 of 10 feces and then *astA*-positive *E. coli* strains were isolated from 6 samples. In case 10 in which norovirus was detected from 20 of 22 feces, the *eae* gene of EPEC or EHEC was detected from 8 of 22 feces and EHEC O26 strains were isolated from 8 of 22 feces. In 7 cases (cases 1, 11, 12, 13, 16, 20, and 21), the pathogenic *E. coli* strains belonging to *astA*-positive *E. coli* and/or EPEC were concomitantly detected with other foodborne bacterial pathogens. In case 1, the *eae* gene of EPEC or EHEC was detected from 4 of 22 feces and the *astA* gene of EAEC was detected in 3 other feces. However, duplex SG-PCR could not detect other virulent genes, including the *stx*1 and *stx*2 genes of EHEC. Then EPEC strains were later isolated from 5 feces and *astA*-positive *E. coli* from 4 other feces. In case 12, the *astA* gene of EAEC was detected in all 5 feces and the *eae* gene of EPEC or EHEC in 2 feces, but duplex SG-PCR could not detect other *E. coli* virulent genes. The subsequent bacteriological examination could not isolate pathogenic *E. coli* among nonpathogenic *E. coli* flora. In case 16, the *C. jejuni* specific gene was detected in 6 of 9 feces and the *astA* gene of EAEC was detected in 5 feces (both genes from 3 feces). *C. jejuni* strains were then isolated from 9 of 14 feces, but we were not able to isolate the pathogenic *E. coli* strain among nonpathogenic *E. coli* flora. In cases 19 to 21 analyzed improved real-time PCR using 8 primers for the detection of 7 main foodborne bacteria and *astA*-positive *E. coli*, *C. jejuni*, EPEC, or *astA*-positive *E. coli* were detected from 1 to 3 fecal samples on the first run and the absence of the other main foodborne bacteria in the analyzed samples was readily confirmed. In case 20, the *eae* gene of EPEC or EHEC was detected from 2 of 5 fecal samples on the first run and the *gyrB* gene of *P. shigelloides* was detected separately from other 2 fecal samples on the second run. Then *P. shigelloides* strains were isolated from 2 feces, but isolation of the EPEC strain was very difficult due to the presence of large nonpathogenic *E. coli* flora in the feces.

In almost all cases, the duplex SG-PCR assay first run detected these causative agents from more than one of the five feces. Then, in almost all cases, the presence of a causative agent (presumed from duplex SG-PCR assay) was confirmed by the results of the final SG-PCR assay run and the bacteriological cultivation of additional feces. These findings confirmed that for foodborne outbreaks duplex SG-PCR is a useful tool for the rapid detection of both single and multiple pathogens.

### 3.3. Quantification of the Causative Agent in 14 Foodborne Outbreak Cases

Figure 2 shows the relationship between CFU and DNA copy of foodborne pathogens using SG-quantitative PCR (qPCR) assay in 71 feces from 14 cases examined by viable cell counting. There was no correlation (*r* ^2^ = 0.1183) between CFU and DNA copy of foodborne pathogens in feces, although almost all pathogens were detected by SG-PCR from feces registering more than 10^3^ CFU/g by viable cell counting. By using SG- qPCR assay combined with DNA extraction using the QIAamp DNA Stool Mini kit, Bibbal et al. [28] reported a significant correlation between CFU and DNA copy of ampicillin-resistant *Enterobacteriaceae* in swine feces. Fu et al. [29] reported a significant correlation between CFU and DNA copy of *Lactobacillus* and total anaerobic bacteria in dog feces but found no correlation between CFU and DNA copy of *C. perfringens*. Although accurate quantifications of foodborne pathogens, including *C. jejuni* and *C. perfringens,* in feces were not completely performed by SG-qPCR in this study, the presence of any foodborne pathogens at more than 10^3^ CFU/g feces was certainly confirmed by melting curve analysis. There are two major problems for these differences. One cause is different sample preparation that was used for CFU from the feces stored in the transport medium and for qPCR using the mass sample collected for virological inspection. Another cause is the approach used to construct the standard curves that were prepared from pure bacterial cultures. These curves do not relate with the “real” situation of a bacterial quantification in a faecal sample and can in part explain the absence of correlation between CFU and DNA copy of foodborne pathogens in faeces.

In our routine bacteriological diagnostic laboratory, we used duplex SYBR Green I PCR assay combined with DNA extraction via QIAamp DNA Stool Mini kit for the detection of foodborne bacteria from 21 foodborne outbreak cases. The causative bacteria were detected in almost all cases in 2 hours or less. The first run was for the detection of 8 main foodborne bacteria and the second run was for the detection of other unusual suspect bacteria. The results proved that for comprehensive and rapid molecular diagnosis in foodborne outbreaks, duplex SG-PCR assay is not only very useful, but is also economically viable for one-step differentiation of causative bacteria in fecal specimens obtained from symptomatic patients. This then allows for effective diagnosis and management of foodborne outbreak.

## Figures and Tables

**Figure 3:**
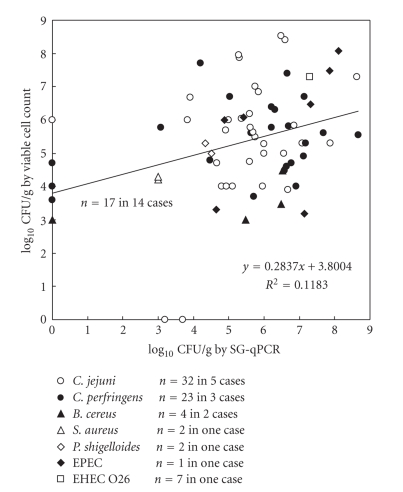
The relationship between CFU and DNA copy of foodborne pathogens in 71 foodborne pathogens-positive feces in 14 foodborne outbreak cases examined by viable cell counting.

**Table 1:** Bacterial strains assayed by SYBR Green I PCR.

Bacterial strains	Sources^e^	PCR results with each primer set (see Table 2)																					
		eae	JMS1	JMS2	LT	STa	EAST-1	aggR-Z	virA	Styinva	yadA-X	PAG	PSG	AB	CC ceuE	hlyA	tdh	trh	AHH1	FemB	ces-TM	SG	GAP
*Escherichia coli* -EPECO55(*eaeA*)	EC-2736^b^	+	−	−	−	−	−	−	−	−	−	−	−	−	−	−	−	−	−	−	−	−	−
*E. coli* -EPEC O153 (*eaeA* and *astA*)	EC-2649^b^	+	−	−	−	−	+	−	−	−	−	−	−	−	−	−	−	−	−	−	−	−	−
*E. coli* -EHEC O26:H11(*Stx1*)	SE-02005	+	+	−	−	−	−	−	−	−	−	−	−	−	−	−	−	−	−	−	−	−	−
*E. coli* -EHEC O157:H7 (*Stx2*)	SE020025	+	−	+	−	−	−	−	−	−	−	−	−	−	−	−	−	−	−	−	−	−	−
*E. coli* -EHEC O157:H7 (*Stx1* and *Stx2*)	SE-02027	+	+	+	−	−	−	−	−	−	−	−	−	−	−	−	−	−	−	−	−	−	−
*E. coli* -ETEC O148 (LT, ST and *astA*)	EC-3515^b^	−	−	−	+	+	+	−	−	−	−	−	−	−	−	−	−	−	−	−	−	−	−
*E. coli* -ETEC O169 (ST and *astA*)	EC-4725^b^	−	−	−	−	+	+	−	−	−	−	−	−	−	−	−	−	−	−	−	−	−	−
*E. coli-* EAEC O111 (*aggR* and *astA*)	EC-4131^b^	−	−	−	−	−	+	+	−	−	−	−	−	−	−	−	−	−	−	−	−	−	−
*E. coli* -EIEC O124:HNM (*virA*)	EA32^a^	−	−	−	−	−	−	−	+	−	−	−	−	−	−	−	−	−	−	−	−	−	−
*Shigella sonnei*	I00031	−	−	−	−	−	−	−	+	−	−	−	−	−	−	−	−	−	−	−	−	−	−
*Salmonella* Enteritidis	Sal-2339	−	−	−	−	−	−	−	−	+	−	−	−	−	−	−	−	−	−	−	−	−	−
*Yersinia enterocolitica* O3/B4	Pa241	−	−	−	−	−	−	−	−	−	+	−	−	−	−	−	−	−	−	−	−	−	−
*Y. pseudotuberculosis* O4b	SP988	−	−	−	−	−	−	−	−	−	+	−	−	−	−	−	−	−	−	−	−	−	−
*Providencia alcalifaciens*	NIID124^C^	−	−	−	−	−	−	−	−	−	−	+	−	−	−	−	−	−	−	−	−	−	−
*Plesiomonas shigelloides*	NIID123^C^	−	−	−	−	−	−	−	−	−	−	−	+	−	−	−	−	−	−	−	−	−	−
*Campylobacter jejuni*	SC 009	−	−	−	−	−	−	−	−	−	−	−	−	+	−	−	−	−	−	−	−	−	−
*Campylobacter coli*	SC 011	−	−	−	−	−	−	−	−	−	−	−	−	−	+	−	−	−	−	−	−	−	−
*Vibrio cholerae* O1	ATCC14035	−	−	−	−	−	−	−	−	−	−	−	−	−	−	+	−	−	−	−	−	−	−
*V. cholerae* O139	NIID63-93^C^	−	−	−	−	−	−	−	−	−	−	−	−	−	−	+	−	−	−	−	−	−	−
*V. cholerae non-* O1	SVP84	−	−	−	−	−	−	−	−	−	−	−	−	−	−	+	−	−	−	−	−	−	−
*V. parahaemoliticus* O3:K6 (tdh)	SVP02	−	−	−	−	−	−	−	−	−	−	−	−	−	−	−	+	−	−	−	−	−	−
*V. parahaemoliticus* O3:K6 (trh)	NIIDK4^C^	−	−	−	−	−	−	−	−	−	−	−	−	−	−	−	−	+	−	−	−	−	−
*Aeromonas hydrophila* O1	ATCC7966	−	−	−	−	−	−	−	−	−	−	−	−	−	−	−	−	−	+	−	−	−	−
*Staphylococcus aureus*	SS 05^e^	−	−	−	−	−	−	−	−	−	−	−	−	−	−	−	−	−	−	+	−	−	−
Emetic *Bacillus cereus*	No.127^e^	−	−	−	−	−	−	−	−	−	−	−	−	−	−	−	−	−	−	−	+	+	−
Enterotoxigenic *B. cereus*	No.1^e^	−	−	−	−	−	−	−	−	−	−	−	−	−	−	−	−	−	−	−	−	+	−
*Clostridium perfringens*	H2^d^	−	−	−	−	−	−	−	−	−	−	−	−	−	−	−	−	−	−	−	−	−	+

Strain kindly donated by K. Sugiyama^a^, Shizuoka Prefectural Institute of Public Health, Shizuoka; J. Yatsuyanagi^b^, Akita Prefectural Institute of Public Health, Akita; M. Tamura and E. Arakawa^c^, ^f^Other strains except for ATCC numbers are our own collections.

**Table 2:** 22 pairs of specific primers for SYBR Green I PCR.

Primer set for duplex PCR	Species and subgroups	Target gene		PCR primers			GenBank accession no.	location	Product size (bp)	*T*_*m*_ values^a^	References
				Name	Forward or revers	primers' sequences (5′ - 3′)					
First run	1 *Escherichia coli*	*eaeA*	∗^*b*^	eae	F2	CATTGATCAGGATTTTTCTGGTGATA	Z11541	899-924	106	83.2 ± 0.2	[14]
	EPEC and EHEC				R	CTCATGCGGAAATAGCCGTTA		1000-979			
	*Staphylococcus aureus*	*femB*	∗	FemB	fw	AATTAACGAAATGGGCAGAAACA	AF106850	277-299	93	80.8 ± 0.3	[15]
					rv	TGCGCAACACCCTGAACTT		370-351			
	2 *Campylobacter jejuni*	*C. jejuni-* specific DNA	∗	AB	F	CTGAATTTGATACCTTAAGTGCAGC	AL111168	381135	86	79.1 ± 0.4	[8]
		DNA			R	AGGCACGCCTAAACCTATAGCT		381185			
	EAEC	*astA*		EAST-1	S	GCCATCAACACAGTATATCC	L11241	63-82	106	84.9 ± 0.6	[16]
					AS	GAGTGACGGCTTTGTAGTCC		168-148			
	3 *Vibrio parahaemoliticus*	*tdh*		Tdh199	F	GGTACTAAATGGCTGACATC	X54341	601-582	251	81.6 ± 0.3	[17]
					R	CCACTACCACTCTCATATGC		351-370			
	Emetic *Bacillus cereus*	*ces*	∗	ces-TM	F	GATGTTTGCGACGATGCAA	DQ360825	8689-8707	65	80.4 ± 0.1	This study
					R	CTTTCGGCGTGATACCCATT		8793-8734			
	4 *Salmonella* spp.	*invA*	∗	Styinva	JHO-2-right	TCGTCATTCCATTACCTACC	M90846	167-186	119	81.3 ± 0.4	[5]
					JHO-3-left	AAACGTTGAAAAACTGAGGA		285-234			
	*Clostridium perfringens*	*cpe*		GAP	11	GGTTCATTAATTGAAACTGGTG	X81849	583-604	154	78.3 ± 0.4	[18]
					12	AACGCCAATCATATAAATTACAGC		712-736			

Second and third runs	5 ETEC (ST)	ST		STa	F	GCTAATGTTGGCAATTTTTATTTCTGTA	M25607	294-321	190	78.5 ± 0.2	[19]
					R	AGGATTACAACAAAGTTCACAGCAGTAA		483-456			
	*Plesiomonas shigelloides*	*gyrB*		PSG	237-F	TTCCAGTACGAGATCCTGGCTAA	AJ300545	237-259	68	83.1 ± 0.2	[13]
					304-R	TGAATCGACACGCCAGAGTTC		304-284			
	6 EAggEC	*aggR*	∗	aggR-Z	F	CAGAATCGTCAGCATCAGCTACA	Z18751	432-454	97	79.5 ± 0.3	This study
					R	GATGCCCTGATGATAATATACGGAA		358-382			
	EIEC & Shigella spp.	*virA*		virA	F	CTGCATTCTGGCAATCTCTTCACA	D26468	1589-1622	215	82.4 ± 0.3	[20]
					R	TGATGAGCTAACTTCGTAAGCCCTCC		1813-1788			
	7 * Aeromonas hydrophila*	*ahh1*	∗	AHH1	F	GCCGAGCGCCCAGAAGGTGAGTT	CP000462	1653360-82	133	89.8 ± 0.4	[21]
					R	GAGCGGCTGGATGCGGTTGT		1653492-73			
	ETEC (LT)	LT		LT	1	AGCAGGTTTCCCACCGGATCACCA	S60731	613-636	132	82.0 ± 0.3	[22]
					2	GTGCTCAGATTCTGGGTCTC		744-725			
	8 *Providencia alcalifaciens*	*gyrB*		PAG	38F	TCTGCACGGTGTGGGTGTT	AJ300547	38-56	73	81.0 ± 0.2	[13]
					110R	ACCGTCACGGCGGATTACT		110-92			
	Enterotoxigenic *B. cereus*	*nheB*	∗	SG	F3	GCACTTATGGCAGTATTTGCAGC	DQ153257	2101-2123	152	82.7 ± 0.4	[23]
					R3	GCATCTTTTAAGCCTTCTGGTC		2252-2231			
	9 *Yersinia enterocolitica*	*yadA*	∗	yadA-X	F	CCAGAACCAATTGCAATGCCT	X13882	1564-1543	100	81.6 ± 0.2	This study
	*Y. pseudotuberculosis*				R	CTTTAAACAGCTTGTTCCAGCCA		1465-1487		81.1 ± 0.3	
	*Campylobacter coli*	*ceuE*	∗	CCceuE	825F	ACGCGCACAAGGCATACTT	X88849	3513-3531	91	77.6 ± 0.3	This study
					915R	CCAGTATTCAGGATCAAGATAAATGATTT		3603-3575			
	10 *Vibrio cholerae*	*hlyA*		hlyA	2272-F	AGCAGCGTGTGGGACAAGA	X51746	2272-2291	71	82.4 ± 0.1	[13]
					2272-F	GCGGACCCTAATGCATCAAT		2342-2323			
	*Vibrio parahaemoliticus*	*trh*		Trh	250-F	GGCTCAAAATGGTTAAGCG	DQ359748	256-274	250	81.1 ± 0.1	[17]
					251-R	CATTTCCGCTCTCATATGC		505-487			
Singl PCR	EHEC (Stx 1)	*Stx1*		JMS1	F	GTCACAGTAACAAACCGTAACA	EF441598	509-488	95	81.1 ± 1.0	[12]
					R	TCGTTGACTACTTCTTATCTGGA		415-437			
Singl PCR	EHEC (Stx 2)	*Stx2*		JMS2	F	CGACCCCTCTTGAACATA	EF441616	140-157	108	81.7 ± 0.3	[12]
					R	GATAGACATCAAGCCCTCGT		247-228			

a: Average ± standard deviation of *Tm* values of 10 tests: b: ∗:new selected or designed primer

**Table 3:** Epidemiological investigations in 21 food—borne outbreaks examined by SG-PCR and bacteriological cultures in Shimane Prefecture, Japan.

Case No.	Date ocurred (day/mo/yr)	Days for examination after occurrence	Infected group	Source of infection (suspected source)	No. of patients/total	No. of examined patients	Causative pathogens	Stool samples (No. of positive/ examined samples)					
								SG-PCR					Isolation
								1st test	2nd test	3rd test	Final test	Total	
1	4-Oct-02	6	School excursion in a mountain area	Stream water^a^	23/33	22	*EPEC O:125, O:166, O:UT *astA-positive *E. coli* O:1, O:UT	1/7	—	—	4/22	7/22	5/22
													
2	03-Sep-03	3	Protective care school	Catering box lunch	22/46	10	*astA*-positive *E. coli* O:18,	1/5	—	—	6/10	6/10	3/10
							O:20, O:114, O:159, O:UT						
							[Norovirus				6/7]		
3	01-Oct-03	2	Celebration in a company	Catering box lunch	437/1354	12	*C. perfringens O:13, O:16	5/5	—	—	7/12	7/12	10/12
4	11-Jun-04	6	Camping group of high school	Grilled meat (beef, bovine intestinal meat)	4/8	4	*C. jejuni*	1/4	—	—	1/8	1/8	5/8
5	12,13-Jun-04	6 ~ 7	9 citizen groups in Chophouse	Grilled meat (beef, bovine intestinal meat)	30/UN	12	*C. jejuni*	4/5	—	—	8/12	8/12	10/12
6	17-Jun-04	5	Cooking practise in a high school	Shelf-cooked lunch (salada mixed chicken)	31/41	20	*C. jejuni	4/5	—	—	12/14	12/14	17/20
7	07-Jul-04	1	Citizen in Chinese restaurant	Fried rice^b^	6/6	6	*B. cereus	1/1	—	—	2/6	2/6	2/6
8	11-Oct-04	3	Sport club in a high school	Shelf-cooked lunch	26/47	6	*C. perfringens O:16, OUT	1/5	—	—	3/6	3/6	4/6
9	5 ~ 7-Nov-04	5 ~ 7	Restaurant	Unknown	5	5	*C. jejuni*	2/5	—	—		2/5	2/5
10	Unknown	Several days (19-Jur-05)	Nursery	Unknown	24/73	22	*EHEC O26				8/22	8/22	8/22
							[Norovirus				20/22]		
11	28 ~ 30-Sep-05	1 ~ 3	Prisoners in a prison	Shelf-cooked meal^c^	113/600	61	*astA* -positive *E. coli*	—	—	14/14		14/14	41/46
							*C. perfringens* (sporadic case)	1/5					1/46
12	2 ~ 6-Oct-05	1 ~ 5	Elementary and high school children	Unknown (School lunch)	39/94	39	*astA* -positve *E. coli*	5/5	—	—		5/5	IM^f^
							EPEC	2/5					IM
							*A. hidrophila*	1/5					1/16
							*C. jejuni* (sporadic case)	1/5					IM
13	28 ~ 30-May-06	0 ~ 2	Citizens at restaurant	Lunch (pilaf and scrambled agg^d^)	27/34	27	*S. aureus	2/5	—	—		2/5	4/8
							*astA-positve E. coli*	1/5					
14	4-Jul-06	0	Boarder of high school	Catering box lunch	34/51	34	*C. perfringens	5/5	—	—	8/8	8/8	19/50
15	16-Aug-06	1	Citizens at restaurant	Fried rice	15/34	15	*B. cereus	1/4	—	—		1/4	2/4
16	23 ~ 29-Aug-06	2 ~ 8	Boarder of training high school	Supper (contaminated sliced cabbage^e^)	19/43	18	*C. jejuni	3/5	—	—	6/9	8/9	9/14
							*astA* -positve *E. coli*	4/5			5/9		IM
17	2-Sep-06	3	Citizens in Buddhist service	Catering box lunch	14/49	4	*V. parahaemolyticus*	4/5	—	—	4/6	4/6	3/6
18	22-Dec-06	5	Citizens at restaurant	Supper (chiken)	12/12	8	*C. jejuni	3/5	—	—	4/9	4/9	4/10
19	4-Jul-07	6	Citizens at restaurant	Supper (chiken)	7/11	7	*C. jejuni	1/2	—	—		1/2	2/3
20	21-Oct-07	1	Citizens at restaurant	Supper	7/13	7	*EPEC	2/5		—		4/5	IM
							*P. shigelloides*		2/5				2/5
21	29-Nov-07	1	Citizens at restaurant	Supper (raw chiken liver)	8/8	7	*C. jejuni	3/5	—	—	4/7	5/7	4/7
							*astA -positve E. coli*	1/5					1/7

Total								54/93				111/191	160/276
								58.1%				58.1%	58.0%

a: EPEC O : 166, O : UT and *astA* -positive *E. coli* O : 27, O : UT strains were isolated from stream water drunk by patients in case 1., b: *B cereus* was isolated from cooked pork in case 7. c: *astA* genes were detected from 5 food samples in case 11., d: *S. aureus* was isolated from pilaf and scrambled egg in case 13., e: *C. jejuni* specific gene was detected from 5 food samples in case 17. f: Impossible isolation. *: 14 cases examined by SG-qPCR and viable cell count.
